# “mTOR Signaling Pathway”: A Potential Target of Curcumin in the Treatment of Spinal Cord Injury

**DOI:** 10.1155/2017/1634801

**Published:** 2017-06-12

**Authors:** Jingquan Lin, Xue Huo, Xuehong Liu

**Affiliations:** ^1^Department of Histology and Embryology, Medical College, Shaoxing University, Zhejiang, China; ^2^Institute of Neuroscience, Zhejiang University School of Medicine, Hangzhou, China

## Abstract

The purpose of this review is to discuss the possibility of the treatment of spinal cord injury (SCI) with curcumin via regulating the mTOR signaling pathway, which may provide another strong support for curcumin to be a promising medicine applied to the treatment of SCI. Curcumin is termed as a multifunctional targeting therapy drug that regulates the mTOR signaling pathway in the treatment of numerous diseases. Previous research has already revealed that mTOR signaling pathway plays a vital role in prognosis, which involves the axon regeneration and autophagy. This review discusses a potential mechanism that curcumin suppresses the activation of this pathway and ameliorates the microenvironment of axons regeneration which would provide a new way that induces autophagy appropriately.

## 1. Introduction

**Figure 1:**
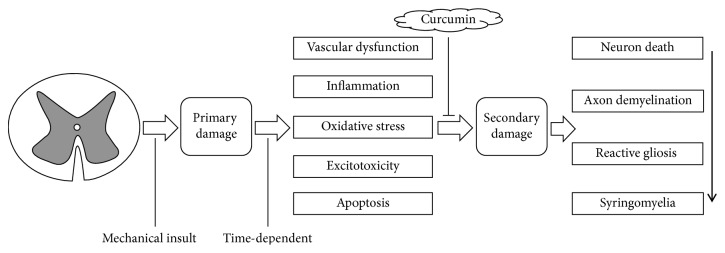
Pathophysiology of SCI and curcumin-induced action in SCI.

Spinal cord injury (SCI) is a devastating central nervous system trauma, which impacts one's health both physically and mentally which caused the family and society to sink into a heavy burden, yet global prevalence and incidence of traumatic spinal cord injury indicates that millions of people have been suffering from SCI in past decades [1]. There are two pathological processes that appear when the spinal cord injury is acute damage; one is called “primary injury,” which is caused by a sharp external force in constriction or laceration; this change is irreversible; the other also was deemed as the most terrible/serious one is “secondary injury,” which is based on the “primary injury”; it results from intricate pathophysiological changes involving vascular dysfunction, inflammation, oxidative stress, excitotoxicity, apoptosis, and so on [2]. Because the “primary injury” is irreversible, the clinical treatments always paid more attention to the secondary injury to inhibit the pathophysiological change and attenuate the secondary injury of post-SCI so as to realize the function of neuroprotection and neuroregeneration. The mammalian target of rapamycin (mTOR) signaling pathway, which influenced cellular functions and metabolism, growth, proliferation, and viability, plays a crucial role in the functional recovery of central nervous system trauma, especially for axon regeneration and autophagy [3–5], which has an extensive link with apoptosis [6–8]. Thus, it will be a potential therapeutic target in the treatment of SCI. Curcumin (Diferuloylmethane, C_21_H_20_O_6_), a natural polyphenol extract from the plant* Curcuma* along with little side-effect as well as being multitarget, was reported to alleviate the secondary injury of post-SCI, which help the rehabilitation of neuron damage after SCI [9] (Figure 1). Moreover,* Curcuma* is a regulator controlling the mTOR signaling pathway in the treatments of diseases [10]. However, few researches relate to curcumin on mTOR signaling pathway after SCI has been reported. In this review, the related mechanism will be discussed.

## 2. Pathophysiology of SCI

The complex pathophysiology of SCI can be described as two stages: primary phase and secondary phase. Nevertheless, most clinical instances show that the secondary stage followed by the primary insult is more significant because the prognosis of SCI is mainly influenced by the secondary injury. Besides, it provides a vital information that might be a promising therapy for preventing deterioration of initial area of injury.

The secondary phase is a delayed and progressive tissue damage following the initial onset of SCI, which occurs over the time course of hours to weeks and is caused by a cascade of biological events. Following the mechanical insult, there is an unavoidable structural destroy and vascular dysfunction that end up with edema, necrosis and ischemia. Vascular dysfunction includes hemorrhage, vasospasm, thrombosis, loss of autoregulation, and disruption of blood spinal cord barrier. These impairments lead to more inflammatory cells across the defective barrier and expansion of the initial damage. The inflammatory response is regarded as an important contribution to secondary damage; inflammatory cells such as macrophages, microglia, T cells, and neutrophils infiltrate the lesion area, which is releasing inflammatory factors such as tumor necrosis factor-*α* (TNF-*α*), interleukin- (IL-) 1*α*, IL-1*β*, and IL-6, and lead to the disorder of the microenvironment of the spinal cord. Besides, infiltrating macrophages, neutrophils, and activated microglia also join the oxidative stress with providing extra sources of free radicals. Free radicals react with the polyunsaturated fatty acid of the cellular membrane yielding to the peroxidation and disorder of the normal phospholipid structure of cellular and subcellular organelle membranes, which significantly contributes to neural tissue damage. In particular, extracellular amino acids, glutamate and aspartate, are increasingly released from disorder cells. The excessive activation of relevant amino acid receptors produces excitotoxicity and leads to the loss of neurons both in necrosis and apoptosis. Apoptosis occurs in neurons, oligodendrocytes, microglia, and astrocytes. Oligodendrocytes are easily subjected to apoptotic loss that contributes to postinjury demyelination [2, 11].

Eventually, all of these processes bring neuron death, axon demyelination, reactive gliosis, and syringomyelia around the lesion. Considering the pathophysiology of SCI, alleviating secondary damage is a key point to the treatment (Figure 1).

## 3. mTOR Signaling Pathway and SCI

mTOR signaling pathway is a promising pharmacological option that is well known for its roles in the process of growing cells, metabolism, and cancer. mTOR, is a core of the signaling networks, which belongs to the phosphoinositide 3-kinase- (PI3K-) related protein kinase (PIKK) family. It assembles into two complexes, mTORC1 and mTORC2, which were composed of its subunit raptor and rictor. Respectively, both contained mLST8 subunit, with distinct inputs and downstream effects [12–15]. Likewise, mTOR has an instrumental role in a great deal of physiological functions of the central nervous system, including the regulation of neuronal cell growth, survival, axonal and dendritic development during differentiation, and synaptic plasticity [16, 17]. Significantly, this pathway is earning novel concern for its role in the traumatic CNS injury repair and regeneration like traumatic brain injury (TBI) and SCI [3–5].

### 3.1. Promoting Axon Regeneration Dependent on Inhibiting Glial Scar Formation via Inhibiting the Pathway after SCI

**Figure 2:**
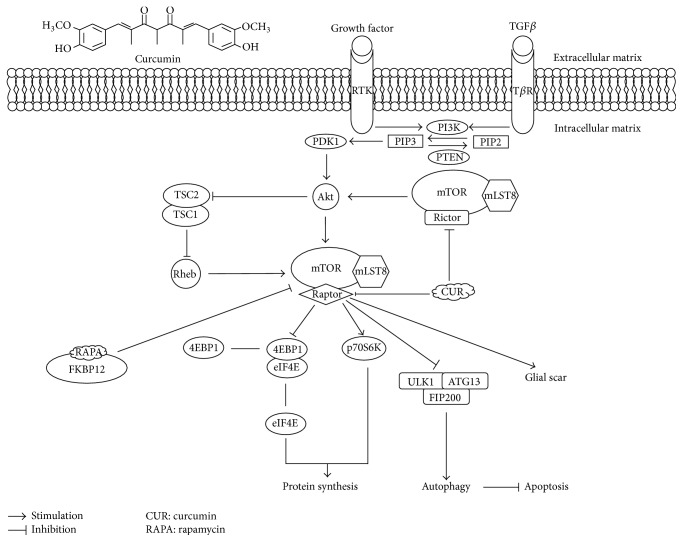
The mTOR signaling pathway and the possibility of curcumin's pharmacological action on the pathway in the treatment of SCI.

Glial scar, an extrinsic environment for axon regeneration, acts like physical and chemical barriers and is mainly composed of reactive astrocytes, with the upregulation of glial fibrillary acidic protein (GFAP) and vimentin, the hallmarks of the gliosis process, and extracellular matrix molecules particularly interact with the chondroitin sulfate proteoglycans (CSPGs), which is degenerated by Chondroitinase ABC (ChABC) allowing axon regeneration and function recovery in SCI [18]. Nevertheless, some findings indicated the diminished spread of the damage by limiting the areas of spinal cord injury, thereby suppressing the axons regeneration and function recovery. MTOR-STAT3 pathway is activated while Nogo receptors have been blocked by Nogo-66. MTOR-STAT3 pathway promotes neural progenitors that differentiate into lineages of astrocytes [19]. The activation of the PI3K/Akt/mTOR signaling pathway involves the formation of glial scar. Rapamycin, as an mTORC1 sensitive allosteric inhibitor, combined with the intracellular 12 kDa FK506-binding protein (FKBP12), suppressed the proliferation of astrocyte and reduced the GFAP positive cells at the damaged area that followed with the progress of axon's reconstruction [20]. Similarly, stimulating the expression of phosphatase and tensin homolog deleted on chromosome 10 (PTEN) inhibits the PI3K/Akt/mTOR signaling pathway and attenuates the formation of glial scar, which may be activated in astrocytes in 3 days after SCI involving gliosis, when decreasing GFAP and vimentin, as well as manipulating CSPGs expression, also improving axon's regeneration into the lesion sites; those enhance the locomotor function after SCI [21]. Interestingly, the expression of CSPGs and the transforming growth factor-*β* (TGF-*β*) are upregulated after SCI through non-Smad-dependent activation of the PI3K/Akt/mTOR signaling pathway, rather than the Smad2/3 signaling pathway [22], but the mechanism still remains unknown. Furthermore, epidermal growth factor receptor (EGR) is performing tyrosine kinase's activity with another term of receptor tyrosine kinase (RTK) within cell membrane, is upregulated in astrocytes after SCI and Ras homolog that are enriched in brain (Rheb), and is essential for mTOR activation through EGR; treatment with rapamycin reduces the expression of GFAP and vimentin, which inhibits the reactive gliosis [23] ( Figure 2).

### 3.2. Promoting Axon Regeneration Depends on Neuron Intrinsic Mechanism via Activating the Pathway after SCI

The constitutive expression activates Rheb in adult neurons after complete SCI enhances axon regeneration cross a ChABC-treated glial scar [24]. However, researches show that it does not eliminate the glial scar instead it made axon regeneration possible for the injured spinal cord. When neural stem cells are transplanted to specific sites in the injured spinal cord, it differentiates into multiple cellular phenotypes including neurons, which extend axons over striking distances and form ample synapses with resident cell [25]. Other researches mostly aim at the PTEN, for example, Liu et al. [26]. It has been reported that before complete spinal cord crush, conditional deletion of the PTEN gene promotes compensatory sprouting of uninjured corticospinal axons; it is also supposed that the regeneration of a large number of injured corticospinal tract (CST) axons crosses the injury site; besides, synapses in spinal segments distal to the injury were reformed by these regenerating CST axons, even though nothing was done to the glial scar formation. What is more, the codeletion of PTEN and suppressor of cytokine signaling 3 (SOCS3) could lead to the change of uninjured CST axons to denervation of the spinal cord [27]. Zukor et al. [28] show that suppression of PTEN's expression with short-hairpin RNA (shRNA) in the neonatal cortex also promotes the regeneration of injured CST axons and even promotes its functional recovery by combining with the treatment of Salmon Fibrin after SCI [29]. Following researches indicate that PTEN deletion shortly after SCI could enhance the regenerative growth of CST axons and motor function [30]. Moreover, the treatment of PTEN's inhibitor peroxovanadium and PTEN's antagonist peptides plays a similar role as PTEN's deletion in the functional recovery [31, 32]. Strikingly, PTEN's deletion promotes regrowth of CST axons even 1 year after chronic SCI [33]. The long-term consequences of conditional genetic deletion of PTEN in the sensorimotor cortex are the only cause of neuronal hypertrophy but no other detectable neuropathology [34]. Yet, there was an age-dependent decline in axon regeneration with PTEN deletion. Compared with young mice, middle-aged to aging mice lose the effects in enhancing axon's regeneration distal to the injury [35]. In addition, mTOR signaling pathway activated by ATP, IL-6, and exercise as well as intermittent hypoxia training also improved the ability of translocating recovery after SCI, followed with the axon's regenerative potential of neurons [36–40].

### 3.3. Inducing Autophagy via Inhibiting the Pathway and Against the Apoptosis after SCI

Autophagy, called “self-eating” refers to an intracellular mechanism involved in the degradation of organelles and long-living proteins lysosome-dependent pathway as well as for cleaning up dysfunctional cellular components; the kinase complex demands to stimulate autophagy in advance. ULK1/Atg13/FIP200 (unc-51-like kinase 1/mammalian autophagy-related gene 13/focal adhesion kinase family-interacting protein about 200 kDa) are directly phosphorylated and suppressed by mTORC1 [15]. LC-3 and Beclin-1 are reliable markers of autophagy and improved autophagy. Rapamycin could decrease the phosphorylation of the p70 S6 kinase (p70S6K) protein and led to higher expression levels of LC-3 II and Beclin-1 in the spinal cord injury along with less TUNEL positive cell; it also acted against the apoptosis and reduced neural tissue damage with locomotor impairment after SCI [20, 41]. Likewise, Simvastatin performed a similar role like Rapamycin in SCI by reducing mTOR protein and p70S6K phosphorylation, while Beclin-1 and LC-3 II are obviously enhanced [42]. Similarly, disturbed autophagy also plays a pivotal role in the pathogenesis of cardiomyopathy associated with uncorrected obesity named cardiomyopathy of obesity [43], and suppression of mTOR may act as a possible approach to hinder pathological cardiac hypertrophy by saving interrupted autophagic flux [44]. More surprisingly, without influencing phosphorylation of Akt, mTOR-independent autophagy inducer trehalose may ameliorate insulin resistance-induced myocardial contractile defect and apoptosis, by means of autophagy associated with dephosphorylation of p38 mitogen activated protein kinase (p38 MAPK) and Forkhead box O 1 (Foxo1) [45]. On the contrary, several investigations have proved that the activation of Akt/mTOR signaling pathway inhibits the excessive autophagy and enhances the recovery following SCI [46, 47]. Hence, only inducing autophagy appropriately could be beneficial to the recovery of SCI. Apoptosis is the process of programmed cell death and entails a series of characteristic cell changes, playing a crucial role in cell death and autophagy. Interestingly, there is an intimate link between apoptosis and autophagy in SCI, and the induction of autophagy could produce neuroprotective effects in acute spinal cord injury in rats via inhibition of apoptosis [48–51] (Figure 2).

## 4. Curcumin Inhibits the mTOR Signaling Pathway

Curcumin, a pleiotropic molecule from* Curcuma longa*, was first found to have antibacterial action in 1949 [52]. Extensive preclinical investigations over the past half century have indicated that curcumin and its analogs overcome the property of low bioavailability, fast metabolism, and bad chemical stability in the clinical application (e.g., C66, MC37, and EF24), which attracted considerable attention holding a gigantic potential in defending and curing several of inflammatory conditions and chronic diseases in cancer [53–55], diabetes and its associated complications [56, 57], diabetic cardiomyopathy [58–60] and diabetic nephropathy [61–63], cardiovascular diseases [64], CNS disorder/trauma [65], and others [66].

At present, curcumin, a well known anticancer medicine entering phase III clinical trials approved by FDA, aims at several signaling pathways and demonstrated the suppression of mTOR signaling pathway [67, 68]. While the treatment of curcumin in SCI related to mTOR signaling pathway remains unreported, the pathway inhibited by curcumin in tumors has been reviewed [9]; 197 proteins were greatly recognized as curcumin binding target from HCT116 Colon Cancer Cell Line, and target is enriched in the nucleus, mitochondria, and plasma membrane; functional analyses show that the cellular protein synthesis downregulation of curcumin can induce autophagy and lysosomal activation [69]. In addition, discovery indicates that curcumin is a direct inhibitor of mTOR, which may remove the association of Raptor with mTOR at low concentration and suppress the binding of Rictor with mTOR at a high concentration [70], but it is not supported by molecular insight into the regulation. Therefore, a much more detailed mechanism about the inhibition of curcumin in mTOR signaling pathway should be further studied (Figure 2).

## 5. Curcumin Inhibits Glial Scar Formation and Apoptosis after SCI

Previous researches have already shown the positive influence of curcumin on the treatment of SCI, and a meta-analysis revealed the neurological recovery effect of curcumin [9]. The expression of GFAP and CSPG proteins is significantly suppressed by curcumin which inhibits the glial scar's formation both intracellularly and extracellularly and ameliorates the microenvironment for axon's regeneration [71–73]. CDGSH iron sulfur domain 2 (CISD2) was regarded as a survival gene like bcl-2 and also known to have role in antiapoptosis, which was showed to exert antiapoptotic effect in neural cells. Curcumin could attenuate the downregulation of CISD2 in SCI [74]. In addition, TUNEL (transferase-mediated dUTP nick end labeling) analysis in some investigations provided a great proof for antiapoptosis activity of curcumin in SCI treatment [75–77] (Figure 2).

## 6. Conclusion

The mTOR signaling pathway is involved in the axon regeneration and autophagy in the pathological process of SCI. While curcumin is also well known as a multiple targets medicine that can inhibit the mTOR signaling pathway in numerous diseases, likewise, the mentioned evidence above indicates that mTOR signaling pathway is a potential therapeutic target of curcumin in the treatment of SCI by ameliorating the microenvironment of axons regeneration which induced autophagy appropriately as well as having little impact on the intrinsic mechanism of the axon regeneration. However, there is still no investigations referring to the curcumin's influence involved in mTOR signaling pathway; thus another research should be done to make the related mechanisms about it clear, and it will provide another strong evidence for the potential utility of curcumin through the treatment of SCI.

## Abbreviations

SCI: Spinal cord injury
mTOR: Mammalian target of rapamycin
TNF-*α*: Tumor necrosis factor-*α*
IL: Interleukin
PI3K: Phosphoinositide 3-kinase
PIKK: PI3K-related protein kinase
TBI: Traumatic brain injury
GFAP: Glial fibrillary acidic protein
CSPGs: Chondroitin sulfate proteoglycans
ChABC: Chondroitinase ABC
FKBP12: The 12 kDa FK506-binding protein
PTEN: Phosphatase and tensin homolog deleted on chromosome 10
TGF-*β*: Transforming growth factor-*β*
EGR: Epidermal growth factor receptor
RTK: Receptor tyrosine kinase
Rheb: Ras homolog enriched in brain
CST: Corticospinal tract
SOCS3: Suppressor of cytokine signaling 3
shRNA: Short-hairpin RNA
p70S6K: p70 S6 kinase
p38 MAPK: p38 mitogen activated protein kinase
Foxo1: Forkhead box O 1
CISD2: CDGSH iron sulfur domain 2.
